# Biopsy Needle Advancement during Bone Marrow Aspiration Increases Mesenchymal Stem Cell Concentration

**DOI:** 10.3389/fvets.2016.00023

**Published:** 2016-03-14

**Authors:** Anne E. Peters, Ashlee E. Watts

**Affiliations:** ^1^Department of Large Animal Clinical Sciences, Texas A&M University, College Station, TX, USA

**Keywords:** cell-based therapy, aspirate, point-of-care, horse, regenerative medicine, MSC

## Abstract

Point-of-care kits to concentrate bone marrow (BM)-derived mesenchymal stem cells (MSCs) are used clinically in horses. A maximal number of MSCs per milliliter of marrow aspirated might be desired prior to use of a point-of-care system to concentrate MSCs. Our objective was to test a method to increase the number of MSCs per milliliter of marrow collected. We collected two BM aspirates using two different collection techniques from 12 horses. The first collection technique was to aspirate BM from a single site without advancement of the biopsy needle. The second collection technique was to aspirate marrow from multiple sites within the same sternal puncture by advancing the needle 5 mm three times for BM aspiration from four sites. Numbers of MSCs in collected BM were assessed by total nucleated cell count of BM after aspiration, total colony-forming unit-fibroblast (CFU-F) assay, and total MSC number at each culture passage. The BM aspiration technique of four needle advancements during BM aspiration resulted in higher initial nucleated cell counts, more CFU-Fs, and more MSCs at the first passage. There were no differences in the number of MSCs at later passages. Multiple advancements of the BM needle during BM aspiration resulted in increased MSC concentration at the time of BM collection. If a point-of-care kit is used to concentrate MSCs, multiple advancements may result in higher MSC numbers in the BM concentrate after preparation by the point-of-care kit. For culture expanded MSCs beyond the first cell passage, the difference is of questionable clinical relevance.

## Introduction

Grafting of autologous bone marrow (BM)-derived cells, as raw BM (1), concentrated BM (2), or BM-derived culture expanded mesenchymal stem cells (MSCs) (3) is used in the horse as a regenerative therapy. Numerous differences exist between each of these autologous therapies. One major difference is the timing of therapeutic application. Both raw BM and concentrated BM can be used for point-of-care therapy, whereas BM-derived MSCs typically undergo a 2- to 4-week culture period (4) prior to implantation.

Concentrated BM, or bone marrow aspirate concentrate (BMAC), is BM that has been processed to increase the concentration of the mononuclear portion, which should also reflect an increase in the concentration of MSCs. The enhanced efficacy of BMAC compared to BM was first described in 1989, with improved healing of delayed union in rabbits (5). Since then, the ability to increase the mononuclear fraction in BMAC compared to BM has been well described in multiple species ranging from an increase of 2.4- to 5-fold in people (6), 7-fold in dogs (7), 3.5-fold in pigs (8), and 5- to 19-fold in the horse (9). The increase in MSC concentration within the mononuclear fraction of BMAC versus unprocessed BM is well accepted and relevant to therapeutic applications. In people with atrophic tibial diaphyseal non-union, treatment success with either BM or BMAC was related to the number of progenitors in the graft (10).

Most BMAC systems for the horse require a minimum of 60 ml of BM (2) and anti-coagulant for the system to separate nucleated cells, and presumably the stem cells, from other BM components. However, it is well known that the contamination of peripheral blood in large volume BM aspirates results in a lower concentration of MSCs per milliliter of BM aspirated. Thus, recommended BM aspirate volumes for patient-side regenerative medicine applications in people are 2–10 ml of BM per aspiration site (11, 12). In the horse, the same effect of MSC dilution in large volume BM aspirates was identified when the numbers of colony-forming unit-fibroblasts (CFU-Fs) in successive 5-ml aliquots of BM were compared (13). Because of the MSC dilution in large volume aspirates, Ishihara et al. tested a specialized needle that allows for collection of BM from several locations within one sternal puncture in anesthetized horses. Use of the multidirectional needle for BM collection allowed them to collect BM aspirates from three different sites within the marrow space without removing, replacing, or advancing the needle beyond their initial placement. Instead of moving the needle to a new space within the sternal marrow space, they twisted a second cannula within the needle, which redirected BM flow from and to each of the three sites. Use of their multidirectional needle resulted in an increased frequency of BMAC samples characterized as “high” in progenitor cells but no differences in the MSC concentration in BM aspirate samples (9).

Maximizing the number of MSCs in the BM aspirate prior to concentration with a device would be important to enhance MSC concentration of the final BMAC product. We wanted to develop a technique in standing and sedated horses that would collect sufficient BM volume for most BMAC systems with a higher concentration of MSCs and a reasonable number of bone puncture sites. The objective of our study was to determine if multiple advancements of the BM biopsy needle from the same sternal puncture would result in a higher number of MSCs in 56-ml of BM compared to collection from a single site (SS). We hypothesized that multiple advancements of the BM needle would result in BM aspirates with higher total nucleated cell count (TNCC). To test this, we collected BM using two different techniques from 12 horses and compared TNCC of BM aspirates, the CFU-F assay of cultured BM, and the number of culture expanded MSCs at the first, second, and third passages from BM collected from a SS versus multiple sites (MS).

## Materials and Methods

### Bone Marrow Collection

All animal procedures were approved by the institution’s animal care and use committee (IACUC 2013-0097). BM aspirations were performed in 12 healthy university-owned horses, and no horses were euthanized for this study.

**Figure 1:**
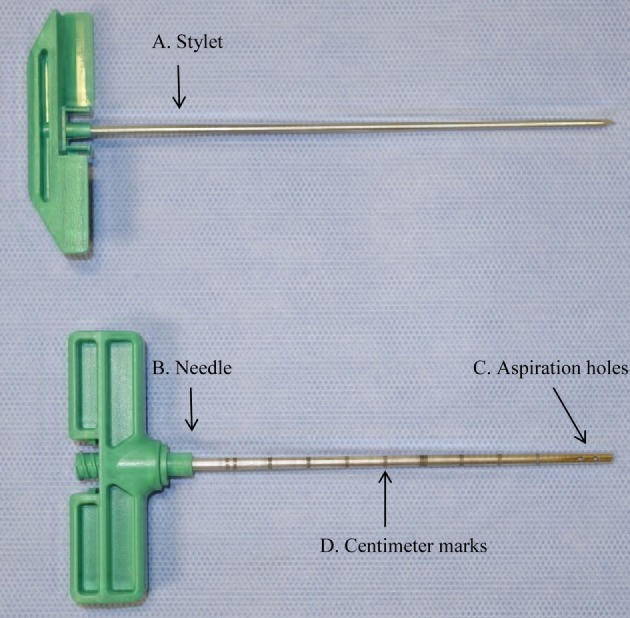
**Photograph of bone marrow (BM) aspiration needle showing (A) stylet, (B) needle, (C) aspiration holes, and (D) centimeter marks used to measure 2 cm distance from the ventral cortical surface of the sternum**.

Bone marrow aspiration was performed from the sternal marrow spaces 4 and 5 using an 11-gage × 10.16 cm BM biopsy needle^1^ with mild sedation of horses (xylazine^2^; 0.4 mg/kg body weight IV) and application of local anesthetic in the subcutaneous tissues (Figure 1). Selection of the site for the fourth and fifth space was made adjacent to the point of the elbow in a square stance and 3–4 cm caudal to that. Collection from the fourth or fifth sternebrae was randomized for the SS and MS aspirations. For the SS collection, the biopsy needle was advanced 2 cm from the ventral cortical surface of the sternum (13), the stylet was removed, and 56 ml of BM was aspirated into a 60-ml syringe pre-filled with 5,000 U of heparin sodium injection.^3^ For the MS collection, a technique similar to that reported by Hernigou et al. was used (10). The BM needle was advanced as for the SS, but only 7 ml of marrow was collected. After the first 7 ml of BM, the needle was rotated 45°, and an additional 7 ml of BM was collected. After the first 14 ml of BM was collected, the stylet was replaced, and the biopsy needle was advanced 5 mm dorsally followed by collection of 7 ml of BM, rotation of the needle, and collection of 7 ml of BM. This advancement and BM collection followed by rotation were repeated two additional times, for a total BM collection of 56 ml and a total distance from the ventral cortical surface of the sternum of 3.5 cm, or 1.5 cm from the initial aspiration site.

### Colony-Forming Unit-Fibroblast Assay

The number of CFU-Fs per milliliter of BM from SS and MS was determined by seeding 1 ml of undiluted and unmanipulated BM to a 10 cm tissue culture dish^4^ (246 cm^2^) containing 9 ml culture medium [Dulbecco’s modified Eagle’s medium (DMEM),^5^ 1 g/l glucose supplemented with 10,000 U/ml Penicillin, 10 mg streptomycin sulfate, 25 μg/ml amphotericin B^6^; 2.5% HEPES buffer^7^; 10 μg/ml human recombinant basic fibroblastic growth factor (see text footnote 7); and 10% fetal bovine serum (FBS)]^8^ and maintained at 37°C in 5% CO_2_, humidified air. Media was exchanged three times per week. Plates were inspected and photographed daily with an Olympus CKX41^9^ microscope. When colonies approached coalescence, media was removed, and the colonies were stained with 3% crystal violet^10^ similar to Franken et al. (14). After plates dried for 24 h, colonies were manually counted without magnification by an observer masked to treatment group.

### Total Nucleated Cell Count

Red blood cell lysis on 10 ml of BM was performed to obtain an initial TNCC. BM was mixed with a sterile red blood cell lysis solution (7.7 mg/ml NH_4_CL^11^; 2.06 mg/ml hydroxymethane–aminomethane^12^; pH 7.2) for 2 min, and then centrifuged at 300 × *g* for 10 min at 4°C. After aspirating supernatant, the cell pellet was resuspended and mixed with lysis solution and centrifuged at 300 × *g* for 10 min at 4°C. The cell pellet was washed with DPBS^13^ and then centrifuged for 5 min at 300 × *g* at 4°C. Cells were resuspended in isolation medium at a volume equal to the original volume of BM aspirate or 10 ml. Samples were diluted 1–10 and a 100-μl sample was used for manual counting of nucleated cells with fluorescein diacetate^14^ and propidium iodide (see text footnote 14).

### Isolation and Expansion of MSCs

Bone marrow aspirate was mixed at a 1:1 ratio with culture medium (described above) and seeded directly onto tissue culture flasks^15^ at 28 ml BM per 175 cm^2^ and maintained at 37°C in 5% CO_2_ humidified air. After 24 h, the same volume of media was added again to the BM media mixture, and half the resultant volume was transferred to a new flask, doubling the number of flasks. Medium was exchanged three times per week. Cells were detached from tissue culture flasks when colonies or monolayers approached 70–80% of confluence with 5 ml Trypsin EDTA^16^ per 175 cm^2^, washed, counted, and reseeded at 5,000 MSCs/cm^2^ until passage 3, when the final expanded MSC number was determined.

### Validation of MSCs

At the conclusion of isolation and expansion, the MSCs from MS and SS were combined and the cell surface marker expression for MHCII,^17^ CD44 (see text footnote 17), CD29,^18^ CD45,^19^ and CD90 (see text footnote 19) were determined for MSCs from each horse using flow cytometry with antibodies that have been previously validated in the horse (15, 16). Cryopreserved cells were thawed, and the concentration was adjusted to make aliquots of one million cells suspended in DPBS. Blocking was performed by 20 min incubation with 10 μl undiluted goat serum. MSCs were pelleted by centrifugation at 400 × *g* for 5 min followed by incubation with antibody dilutions. For MHCII and CD29 and CD44, a 1:100 dilution with conjugated primary antibody was used. For CD90 and CD45, a 1:400 dilution was used with primary and conjugated secondary antibody. Unstained MSCs were used as controls.

Multipotency was tested for SS and MS combined MSCs from all 12 horses and reported as positive or negative. Tri-lineage differentiation was induced by techniques that have been described previously (17–19). For chondrogenic differentiation, aliquots of MSCs (500,000) were centrifuged at 300 × *g* for 5 min to pellet the cells. To induce chondrogenic differentiation, supernatant was aspirated, and 1 ml of chondrogenic induction media [DMEM^20^ 4.5 g/l glucose, supplemented with 10,000 U/ml Penicillin, 10 mg streptomycin sulfate, 25 μg/ml amphotericin B; 2.5% HEPES buffer; 0.2% transforming growth factor β,^21^ 301.89 μg dexamethasone,^22^ 50 μg/ml l-ascorbic acid (see text footnote 22), 40 μg/ml proline (see text footnote 22), 1% ITS+ premix (see text footnote 7), and 1% FBS] was added on top of the pellet. Media was exchanged three times per week for 21 days. Pellets were fixed in 4% formaldehyde (see text footnote 22) for 10 min followed by routine processing, embedding, sectioning, and staining with toluidine blue (see text footnote 22).

Adipogenic differentiation of MSCs was induced by seeding a 10-cm tissue culture dish with 1,000 MSCs/cm^2^. At 70% confluence, existing media was removed and replaced with adipogenic induction media [DMEM Ham’s/F12 1:1,^23^ supplemented with 10,000 U/ml penicillin, 10 mg streptomycin sulfate, 25 μg/ml amphotericin B, 5% rabbit serum (see text footnote 21), 33 μM/l biotin (see text footnote 22), 17 μM pantothenate (see text footnote 22), 1 μM/l insulin (see text footnote 22), 1 μM/l dexamethasone, 225 μl isobutylmethylxanthine (see text footnote 22), 89 μl rosiglitazone (see text footnote 22), and 3% FBS] for 3 days. Media was replaced with adipogenic maintenance media (adipogenic induction media without isobutylmethylxanthine and rosiglitazone) for an additional 3 days followed by staining with oil red O (see text footnote 22).

For osteogenic differentiation, MSCs were seeded on 10 cm culture dishes at 1,000 MSCs/cm^2^. At 70% confluence, media was exchanged for osteogenic induction media [DMEM Ham’s/F12 1:1, supplemented with 10,000 U/ml penicillin, 10 mg streptomycin sulfate, 25 μg/ml amphotericin B, 20 nM/l dexamethasone, 50 μg/ml ascorbic acid, 10 μM/l glycerophosphate (see text footnote 22), and 10% FBS], and cultures were maintained for 14 and 21 days. Media was exchanged three times per week. Cells were stained with 2% alizarin red (see text footnote 22).

### Statistical Analysis

Data were imported to a commercially available statistical analysis program (Statistix 9)^24^ and tested for normality. Paired data for each horse were compared using Wilcoxon-signed-rank test or paired *t*-test as appropriate for data normality. Differences were considered significant when *p* ≤ 0.05 (one-tailed).

## Results

### BM Collection

Twelve female horses ranging in age from 7 to 16 years were used for BM collection. They were all of Quarter Horse type. BM collection went well without incident. Horses were under our care for the following 6 months. No adverse events were recorded.

### CFU-F Assay

**Figure 2:**
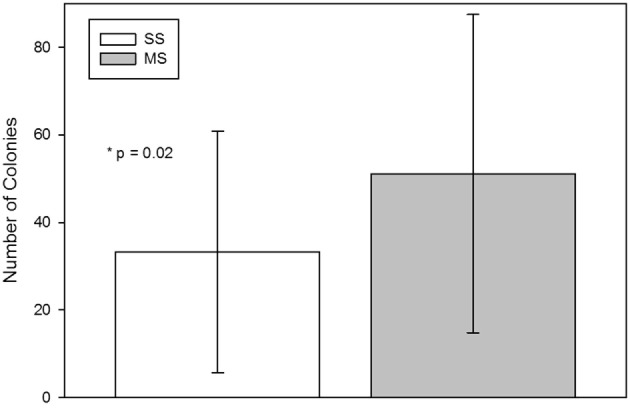
**Colony-forming-unit-fibroblast (CFU-F) assay from bone marrow aspirates using a single site (SS) or a multiple site (MS) technique during bone marrow aspiration (mean, standard deviation)**. There were significantly more CFU-Fs from bone marrow collected with the MS technique (*p* = 0.02, paired *t*-test).

The number of CFU-F from the MS technique was significantly (mean; SD) higher than from the SS technique [SS, 33 (27.6); MS, 51 (36.4); *p* = 0.02; Figure 2].

### Total Nucleated Cell Count

**Figure 3:**
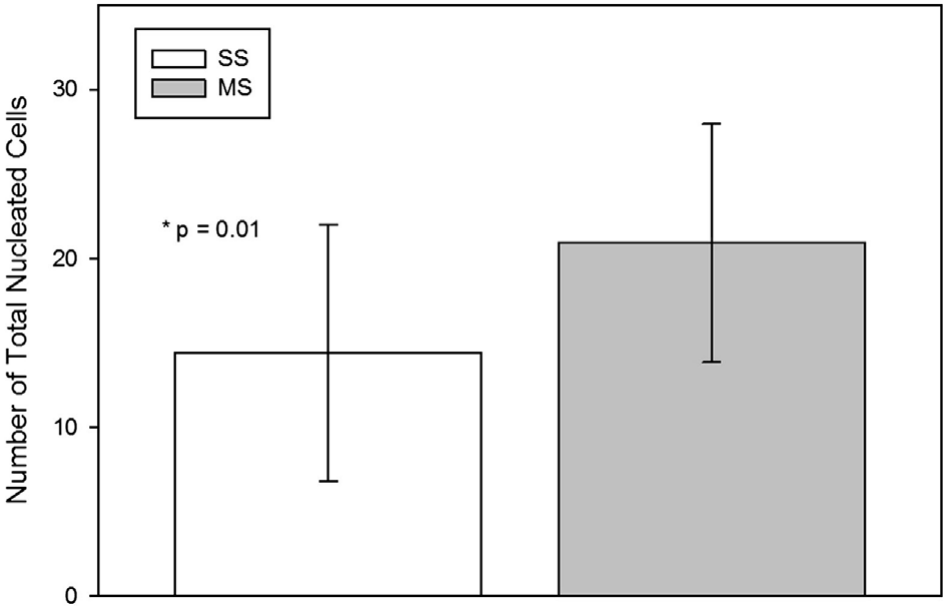
**Total nucleated cell count (mean, standard deviation) per milliliter of raw bone marrow aspirated using a single site (SS) or a multiple site (MS) technique during bone marrow aspiration**. There were significantly more nucleated cells in BM collected with the MS technique (*p* = 0.01, paired *t*-test).

The TNCC from the MS technique was significantly (mean; SD) higher than from the SS technique [SS 14.4 × 10^6^ (7.6 × 10^6^); MS 20.9 × 10^6^ (7.0 × 10^6^); *p* = 0.01; Figure 3].

### Isolation and Expansion of MSCs

**Figure 4:**
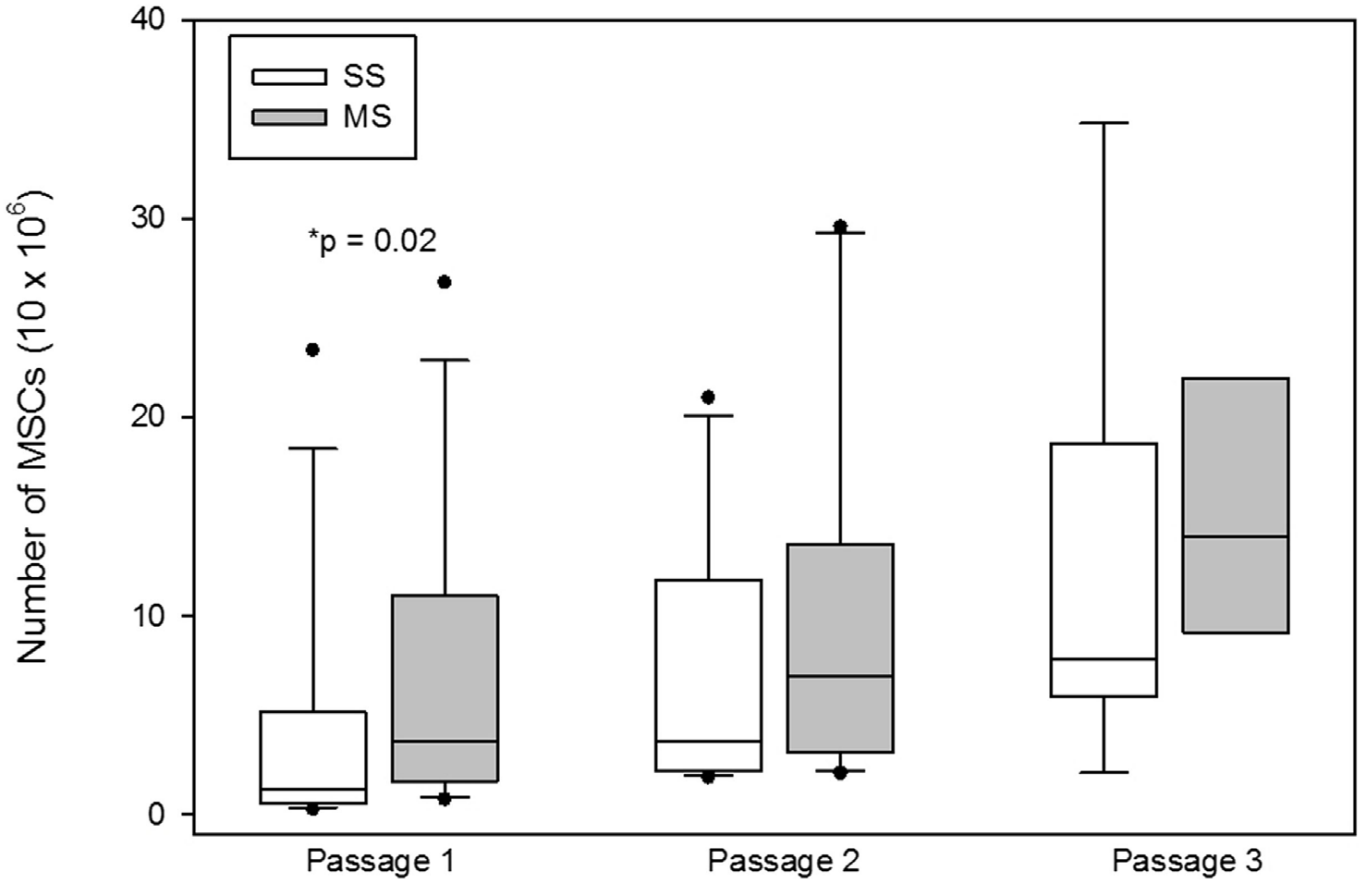
**Total number of mesenchymal stem cells (MSCs; mean, standard deviation) at the each passage from bone marrow aspirates using a single site (SS) or a multiple site (MS) technique during bone marrow (BM) aspiration**. There were significantly more MSCs from BM collected with the MS technique (*p* = 0.02, Wilcoxon-signed rank test). The number of MSCs at passages 2 and 3 were not significantly different between SS and MS techniques (*p* = 0.1; *p* = 0.2).

The number of MSCs at the first passage was significantly (median; interquartile range) higher at the first passage with MS compared to SS aspiration [SS 1.5 × 10^6^ (0.52 × 10^6^–5.16 × 10^6^); MS 3.15 × 10^6^ (1.65 × 10^6^–10.98 × 10^6^); *p* = 0.02; Figure 4], at the second passage [SS 4.95 × 10^6^ (2.17 × 10^6^–11.77 × 10^6^); MS 7.0 × 10^6^ (3.16 × 10^6^–13.57 × 10^6^); *p* = 0.1; Figure 4], and at the third passage [SS 7.84 × 10^6^ (5.95 × 10^6^–18.7 × 10^6^); MS 15.45 × 10^6^ (9.15 × 10^6^–21.97 × 10^6^); *p* = 0.2; Figure 4], there was no significant difference in the number of cells between the two aspiration techniques.

### Validation of Cell MSCs

The cell surface marker profile was consistent with previous reports in the horse (15). MSCs from 8 of 12 horses were negative for MHCII expression, 11 of the 12 were positive for CD90 and CD29 and 12 of 12 were negative for CD45. MSCs from one horse displayed mixed expression of both CD90 and CD29.

**Figure 5:**
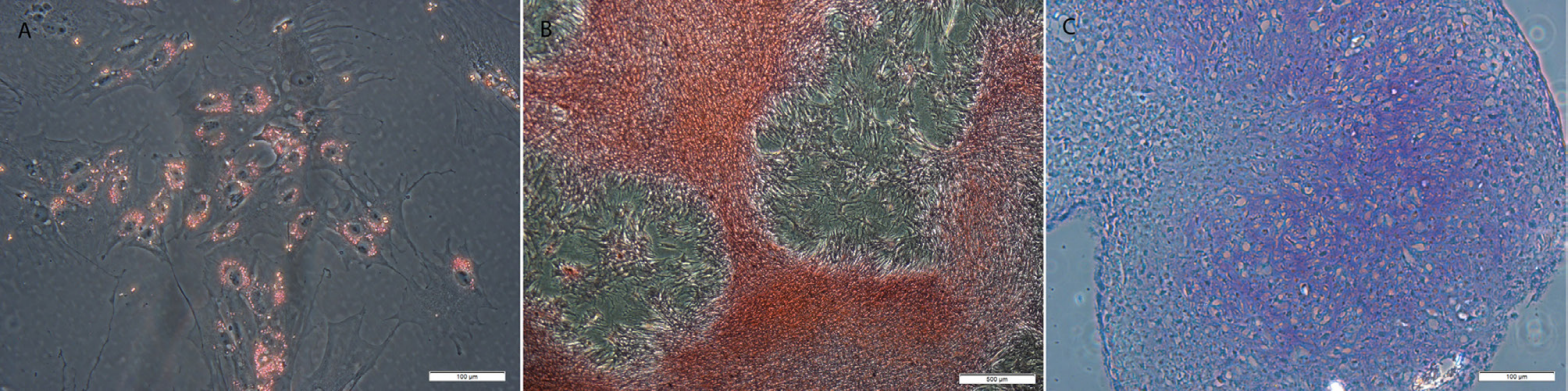
**Examples of MSCs after (A) adipogenic, (B) osteogenic, and (C) chondrogenic induction and staining with resulting in lipid, calcium, and proteoglycan accumulation**. Original magnification 200× **(A,C)** and 40× **(B)**; scale bar = 100 μm **(A,C)** or 500 μm **(B)**.

All MSCs were positive for tri-lineage differentiation (Figure 5).

## Discussion

Our objective was to determine whether multiple advancements (MS) within the same sternal puncture would result in a greater number of MSCs in a 56-ml BM aspirate compared to collection of 56 ml BM from a single site (SS). We found a significantly higher number of nucleated cells in the BM aspirate, and after culture a significantly higher number of CFU-Fs and a significantly higher number of MSCs at the first passage from MS compared to SS aspirates. The increase in CFU-F number and MSC number at the first passage supports that the increased TNCC was due at least in part to a higher number of MSCs in the BM aspirate. At later passages, the total number of MSCs was not different between SS and MS. We think this is because of the rapid growth (4) of MSCs that once isolated, quickly overcome differences in initial MSC concentration. This information is important to the equine veterinarian using BM in patient-side BMAC applications, where large BM volumes are required (most BMAC kits use a total BM volume of 60 ml) and a higher concentration of MSCs in the BM aspirate prior to concentration in the kit could result in higher MSC numbers in the final BMAC product.

Estimates of MSC concentration in BM are 0.001–0.01% (20). This is probably an underestimate of the MSC concentration because it was determined using aspirate volumes of 10–30 ml of BM aspirate per site (20). Regardless, the total number of MSCs in the aspirate, combined with the efficiency of the BMAC system, ultimately determines the number of MSCs in the BMAC product. Therefore, increasing the concentration of MSCs in the BM aspirate is a step the clinician can take to maximize MSC number in the final BMAC preparation.

To maximize progenitor concentration using recommendations from human medicine of 2 ml BM per aspiration site as suggested by Muschler et al. (12) and 10 ml BM per aspiration site as suggested by Hernigou et al. (21) 30 and 6 separate aspirations, respectively, would be required to collect 60 ml, which is the typical BM volume for currently available veterinary BMAC kits (3, 7, 9). To minimize peripheral blood contamination with a more reasonable number of bone punctures, Thoesen et al. collected 7 ml of BM from five separate aspiration sites from each humerus in dogs (7). To achieve the same goal with even fewer cortical bone penetrations in the horse, another group described a specialized needle that increased the frequency of BMAC samples characterized as “high” in progenitor cells but resulted in no differences in MSC concentration of BM aspirates (9). We think our MS technique is similar to the multidirectional needle, in that BM is collected from different sites within a single sternal puncture; however, in contrast to Ishihara et al., we found significant differences in MSC concentration in the original BM aspirate. Our MS technique with multiple manual advancements of the biopsy needle might be more efficient at entering naive lacunae than the multidirectional needle. Increased efficiency could have resulted in less peripheral blood contamination and a higher MSC concentration in the BM aspirate even without a BMAC processing step.

We think the 3 advancements of 5 mm each of the biopsy needle are responsible for the increased MSC concentration due to reduced peripheral blood contamination. Histological specimens after BM collection have demonstrated a minimal number of lacunae are penetrated during BM aspiration and each lacuna is filled with hemorrhage (13). Without biopsy needle advancement, we think continued aspiration from the same site simply collects hemorrhage from the penetrated lacunae, rather than additional progenitors as has been demonstrated previously (13). By advancing the biopsy needle between aspirations, new bony trabeculae are penetrated, allowing BM collection of MSC-rich BM rather than BM hemorrhage. Additionally, the 45° twist of the needle at each site of aspiration repositions the holes along the side of the needle and could also communicate with new lacunae.

One concern about the MS technique might be the increased risk of cardiac puncture with multiple advancements of the biopsy needle. The dorsoventral dimension of the marrow space, not including the cortical bone thickness, within the fourth and fifth sternebrae of the horse is approximately 6.0 and 4.5 cm, respectively (13). Routine technique for the aspiration of BM from the equine sternum is to advance the biopsy needle to a depth of 2-cm from the ventral cortex (13). This depth would leave a minimum additional 4 and 2 cm, respectively, of marrow space that the needle could be advanced into prior to contacting the dorsal cortex. Our MS technique results in an additional advancement of 1.5 cm after the initial placement, 2 cm from the ventral surface of the sternum. Therefore, with accurate placement of the biopsy needle on midline, and careful needle advancement in 5 mm increments, we think the risk of penetration of the dorsal cortex is very low. Certainly, we did not have any complications in this group of 12 horses where we utilized this technique and we have not had complications on the approximately 120 clinical horses where we used the technique for BM aspiration.

A limitation of our study is that we only have indirect proof of increased MSC number with the MS technique. However, the increased TNCC in MS samples is unlikely to be caused by an increase in the white blood cell population, but rather an increase in progenitor populations, including MSCs. This is supported by the increase in numbers of colonies in the CFU-F assay from MS samples that reflect a higher concentration of MSCs in the original aspirate and the higher number of MSCs from MS at the first passage (22).

In conclusion, we tested two techniques for collection of 56 ml of BM from the equine sternum. In our 12 horses, use of the MS technique resulted in a higher concentration of MSCs in the BM aspirate as compared to the SS technique. The higher concentration of MSCs was determined by comparing the TNCC and the CFU-F ability of BM aspirates as well as the total number of MSCs at the first passage during culture expansion. Based on our findings, the MS technique for BM aspiration might be useful in same day applications such as BMAC or BM grafting if an increased number of MSCs in the BMAC product is desired. Future studies could test whether increased MSC concentration using the MS technique causes a greater treatment effect of BMAC compared to using BM from the SS technique. The MS technique may also be useful if the clinician wanted to use very early passage MSCs.

## Author Contributions

AP participated in study design, performed laboratory work, performed statistical analysis, and drafted the manuscript. AW conceived, designed, and coordinated the study, and assisted in drafting and revising the manuscript. All authors contributed to data interpretation, and all authors read and approved the final manuscript.

## Conflict of Interest Statement

The authors declare that the research was conducted in the absence of any commercial or financial relationships that could be construed as a potential conflict of interest.

## Footnotes

^1^Ranfac Corporation, Avon, MA, USA

^2^VetOne®, Boise, ID, USA

^3^Sagent Pharmaceuticals, Schaumburg, IL, USA

^4^Bio Basic, Amherst, NY, USA

^5^DMEM 1 g/l glucose Mediatech, Manassas, VA, USA

^6^Mediatech, Inc., Manassas, VA, USA

^7^Corning, Corning, NY, USA

^8^HyClone, Thermo Scientific, Logan, UT, USA

^9^Olympus, Center Valley, PA, USA

^10^Crystal Violet Sigma-Aldrich, St. Louis, MO, USA

^11^Avantor Performance Materials, Macron Fine Chemicals, Center Valley, PA, USA

^12^Sigma, St. Louis, MO, USA

^13^Lonza, Walkersville, MD, USA

^14^EMD Chemicals, Inc., Calbiotech, San Diego, CA, USA

^15^175 cm^2^ Polystyrene Tissue Culture Flask with Vented Caps Corning, Corning, NY, USA

^16^Trypsin EDTA, Corning, Corning, NY, USA

^17^Bio-Rad, Raleigh, NC, USA

^18^Beckman Coulter, Brea, CA, USA

^19^VMRD Inc., Pullman, WA, USA

^20^DMEM 4.5 g/l glucose Mediatech, Manassas, VA, USA

^21^Life Technologies, Grand Island, NY, USA

^22^Sigma-Aldrich, St. Louis, MO, USA

^23^DMEM F12 Hams 1:1 mix, Mediatech, Manassas, VA, USA

^24^Statistix Analytical Software, Tallahassee, FL, USA

## Supplementary Material

Table S1 **Colony-forming unit-fibroblasts (CFU-Fs) expressed in number of colonies per milliliter of raw bone marrow aspirates and total nucleated cell count (TNCC) per milliliter of raw bone marrow expressed as number of cells ×10^6^ from single site (SS) and multiple site (MS) techniques**.
