# Supplementary material for: Biopsy Needle Advancement during Bone Marrow Aspiration Increases Mesenchymal Stem Cell Concentration
Source: Front Vet Sci. 2016 Mar 14;3:23. doi: 10.3389/fvets.2016.00023 (PMC4789557; doi:10.3389/fvets.2016.00023)
Supplement: Table S1 — Colony-forming unit-fibroblasts (CFU-Fs) expressed in number of colonies per milliliter of raw bone marrow aspirates and total nucleated cell count (TNCC) per milliliter of raw bone marrow expressed as number of cells ×106 from single site (SS) and multiple site (MS) techniques. [file Table_1.PDF]

Horse	CFU-F<br>SS	CFU-F<br>MS	TNCC<br>SS	TNCC<br>MS
1	38	33	15.2	13.5
2	9	57	11.6	20.8
3	10	14	10.6	30.2
4	9	58	14.1	29.5
5	72	61	31.6	28.4
6	5	9	21.6	21.9
7	27	34	6.6	17.6
8	38	63	9.9	14.5
9	67	42	19.2	19
10	84	151	19.9	19.9
11	10	59	5.3	27.8
12	30	33	7.5	7.8
